# A novel mutation in *SORD* gene associated with distal hereditary motor neuropathies

**DOI:** 10.1186/s12920-024-01940-5

**Published:** 2024-06-24

**Authors:** Xiaoqin Yuan, Shanshan Zhang, Huifang Shang, Yufeng Tang

**Affiliations:** 1 Department of Neurology, School of Medicine, Mianyang Central Hospital, University of Electronic Science and Technology of China, Mianyang, Sichuan 621000 China; 2 Department of Neurology, West China Hospital, Sichuan University, Chengdu, Sichuan 610041 China

**Keywords:** SORD, Distal hereditary motor neuropathy, Homozygous mutation, Genetic diagnosis, Mutation spectrum

## Abstract

**Background:**

Distal hereditary motor neuropathy (dHMN) is a heterogeneous group of hereditary diseases caused by the gradual degeneration of the lower motor neuron. More than 30 genes associated with dHMN have been reported, while 70–80% of those with the condition are still unable to receive a genetic diagnosis.

**Methods:**

A 26-year-old man experiencing gradual weakness in his lower limbs was referred to our hospital, and data on clinical features, laboratory tests, and electrophysiological tests were collected. To identify the disease-causing mutation, we conducted whole exome sequencing (WES) and then validated it through Sanger sequencing for the proband and his parents. Silico analysis was performed to predict the pathogenesis of the identified mutations. A literature review of all reported mutations of the related gene for the disease was performed.

**Results:**

The patient presented with dHMN phenotype harboring a novel homozygous variant c.361G > C (p.Ala121Pro) in *SORD*, inherited from his parents, respectively. A121 is a highly conserved site and the mutation was categorized as “likely pathogenic” according to the criteria and guidelines of the American College of Medical Genetics and Genomics (ACMG). A total of 13 published articles including 101 patients reported 18 SORD variants. Almost all described cases have the homozygous deletion variant c.757delG (p.A253Qfs*27) or compound heterozygous state of a combination of c.757delG (p.A253Qfs*27) with another variant. The variant c.361G > C (p.Ala121Pro) detected in our patient was the second homozygous variant in SORD-associated hereditary neuropathy.

**Conclusion:**

One novel homozygous variant c.361G > C (p.Ala121Pro) in *SORD* was identified in a Chinese patient with dHMN phenotype, which expands the mutation spectrum of *SORD*-associated hereditary neuropathy and underscores the significance of screening for SORD variants in patients with undiagnosed hereditary neuropathy patients.

## Introduction

Distal hereditary motor neuropathy (dHMN) is a heterogeneous group of hereditary diseases caused by the gradual degeneration of the lower motor neurons without notable sensory involvement [1]. The typical phenotype of dHMN is muscle weakness and atrophy, which starts in the distal lower limbs in childhood or adulthood [2]. The axonal type of Charcot-Marie-Tooth disease type 2 (CMT2) is characterized by limited sensory neuropathy, and dHMN sometimes share similar clinical features. Additionally, it overlaps with CMT2 in causative genes, including *GARS, IGHMBP2, AARS, DYNC1H1*, and *HARS* [3–5]. More than 30 genes associated with dHMN have been reported [6], while 70–80% of those with the condition are still unable to receive a genetic diagnosis [7]. In recent years, mutations in the sorbitol dehydrogenase (*SORD*) gene were found to be a prevalent cause of recessive dHMN, responsible for about 10% of undiagnosed cases of dHMN and CMT2 [8]. In this study, we reported a novel homozygous mutation c.361G > C (p.Ala121Pro) of *SORD* in a Chinese patient presented with dHMN and evaluated detailed clinical and electromyographic data. At last, a literature review of all reported variants of the *SORD* gene for the disease was performed.

## Materials and methods

### Subject

The proband, a 26-year-old man from Mianyang City in Sichuan Province, Southwest of China, was referred to our hospital because of progressive weakness in the lower limbs. The clinical presentation, laboratory data, and electrophysiological tests were collected.

### Whole exome sequencing

Peripheral blood samples were collected from the patient and his parents, and genomic DNA was extracted by using a standard kit (Qiagen, Germany). At first, the 17p11.2 copy number variation was screened. Subsequently, whole-exome sequencing (WES) was conducted by using the Agilent SureSelectTM Human All Exome V6 kit (Agilent Technologies Inc, Canada) on an Illumina HiSeq2500 (Illumina, San Diego, CA, USA), and the variants were filtered (≤ 1%) according to their frequency in reference population databases.

### Bioinformatics analysis

The variants were annotated by multiple databases, including HGMD (http://www.hgmd.cf.ac.uk/ac/index.php) [9], OMIM (https://www.omim.org/) [10], gnomAD (http://gnomad.broadinstitute.org/) [11], ExAC (http://exac.broadinstitute.org/) [12], and ClinVar (https://www.ncbi.nlm.nih.gov/clinvar/) [13], and variants with an allele frequency ≤ 1% were selected. To predict the potential pathogenicity of the variant, several *in silico* pathogenicity prediction tools, including PolyPhen-2 (http://genetics.bwh.harvard.edu/pph2/index.shtml) [14], NetGene2 (https://services.healthtech.dtu.dk/service.php?NetGene2-2.42) [15], and BDGP (http://www.fruitfly.org/seq_tools/splice.html) [16] were used. To predict the structural changes in protein by substitution of an amino acid, the Missense3D tool was used. (http://missense3d.bc.ic.ac.uk/~missense3d/) [17]. Finally, candidate variants were interpreted according to the American College of Medical Genetics and Genomics (ACMG) (Richards et al., 2015) [18].

### Sanger sequencing

Sanger sequencing was performed using an ABI 3730XL DNA analyzer (Applied Biosystems, Waltham, MA) following the manufacturer’s protocol to validate the filtered variants detected by WES and for segregation analysis.

### Review of the literature

We reviewed all the previously reported cases of CMT or dHMN associated with SORD mutations, with particular emphasis on clinical, electrophysiologic, pathologic, and genetic data. The search strategy included the following: PubMed search with “SORD,” “Charcot-Marie-Tooth” or “Distal hereditary motor neuropathies” used as keywords for articles.

## Results

### Clinical information

The male proband developed progressive weakness and atrophy in the lower limbs at the age of 11 and had a duration of 15 years. He had normal developmental history. He also complained of numbness besides weakness. His parents were not consanguineous and did not have any neurological disorders. A neurological examination was performed on the patient in May 2022, when he was 26 years old. The examination revealed bilateral distal weakness, in the upper limbs (graded as 4+) and lower limbs (graded as 4). Apparent muscle atrophy of lower limbs and pes cavus were noticed. Deep tendon reflexes showed the brisk knee jerk and absence of ankle reflex and Babinski’s sign. The pinprick and temperature sensation were normal. Mild fasciculations were observed in the thighs.

Laboratory tests revealed that blood routine, routine urine, liver and kidney function, and creatine kinase were normal. There were no abnormalities in fasting blood glucose, glycosylated hemoglobin, thyroid function, antibodies, tumor markers, ANA/ENA/ANCA antibody profiles, vitamin B12, and intrinsic factor antibodies. Hepatitis B markers, HIV, and TPPA, were negative. Head MRI showed no remarkable abnormalities. Nerve conduction studies showed reduced compound muscle action potential (CMAP) amplitude in both lower limbs. Motor and sensory nerve conduction velocity and sensory nerve action potentials were in the normal range. Needle electromyography revealed chronic denervation in the proximal and distal muscles (Table 1).

**Table 1:** Motor nerve conduction(MNC) and sensory nerve conduction (SNC) values of the proband

Nerve	Lat(ms)	Amp(mV)	CV(m/s)
(a) motor			
**Peroneus right**			
Ankle-EDB	5.15	0.5	
Fib head-Ankle	11.46	0.6	48
**Peroneus left**			
Ankle-EDB	4.13	1.5	
Fib head-Ankle	10.35	1.7	44
**Tibialis right**			
Ankle-AH	4.81	1.2	
Pop fossa-Ankle	13.19	0.6	48
**Tibialis left**			
Ankle-AH	5.79	3.6	
Pop fossa-Ankle	14.77	2.2	45
(b) sensory			
**Suralis right**			
Calf-Ankle	2.42	16.7	48
**Suralis left**			
Calf-Ankle	2.69	20.1	47
**peroneus superficialis right**			
Lat leg-Ankle	2.44	25.1	49
**peroneus superficialis left**			
Lat leg-Ankle	2.48	14.6	50

### Genetic data

**Fig. 1:**
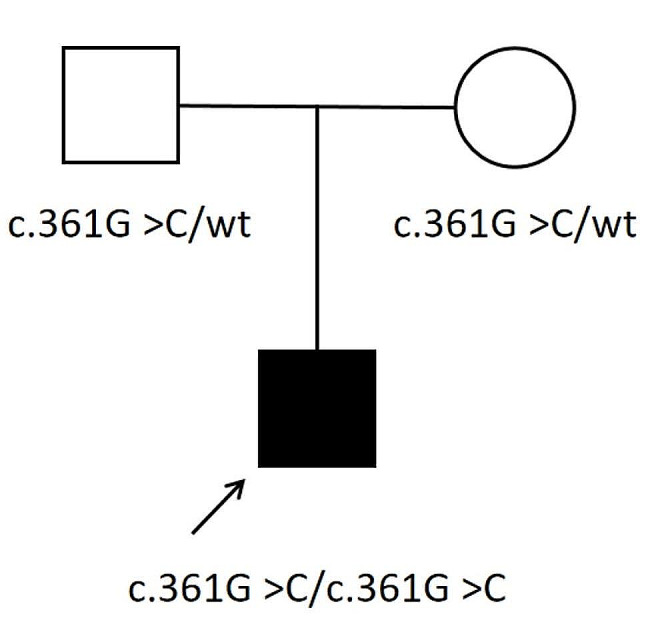
Pedigree of the Chinese family with dHMN. The arrow indicates the proband

**Fig. 2:**
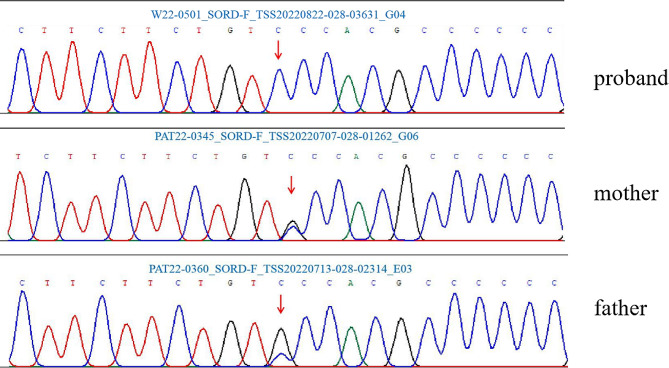
Chromatograms of the SORD gene. The patient carried the homozygous c.361G > C mutation and his parents harbored the heterozygous c.361G > C mutation (arrows)

**Fig. 3:**
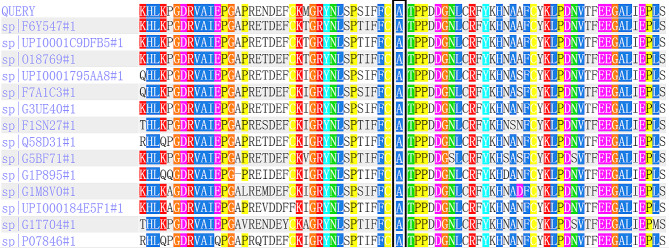
Analysis of conserved sequences showed a total of 75 amino acids surrounding the mutation position (marked with a black box)

**Fig. 4:**
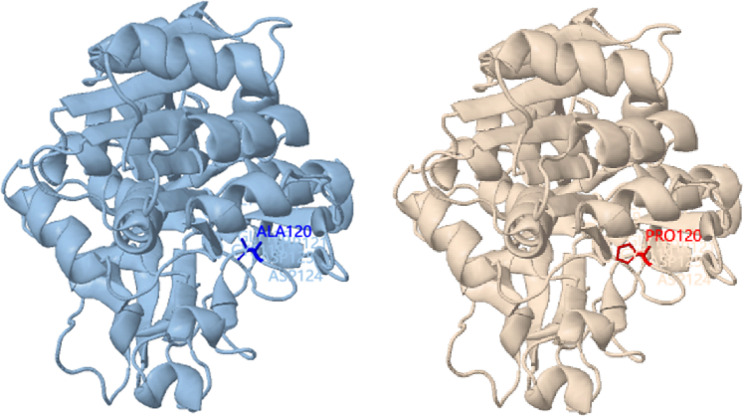
Three-dimensional(3D) structures of the SORD wild-type protein (**A**) and the mutant protein (**B**). The blue represents the ALA residue. The red represents the Pro residue

Whole exome sequencing of the proband detected the homozygous variant c.361G > C (p.Ala121Pro) in the *SORD* gene. The variant is a missense mutation in the coding region of the *SORD* gene (NM_003104.6), which was not reported in the 1000 Genomes Project, Exome Aggregation Consortium (ExAC), and the Genome Aggregation Database (gnomAD). The pedigree of the family is shown in Fig. 1. Sanger sequencing and co-segregation were further conducted on the family, including the proband and unaffected parents (Fig. 2). Conserved sequence analysis suggested the variant is located in a highly conserved sequences across various mammalian species (Fig. 3). The variant c.361G > C was predicted to be possibly damaging with a score of 0.822 by PolyPhen-2. The MutationTaster was used to predict the pathogenicity of the mutation, and it showed that the variation was disease-causing, with a PhyloP score of 5.277 and a PhastCons score of 1. The impact of the p.Ala121Pro mutation on 3D structure of the protein was detected by the Missense3D tool, which showed structural damage of buried proline. The buried proline was predicated to lead to secondary structure changes. The protein structure of *SORD* wild-type and mutant-type were constructed by the Missense 3D tool (Fig. 4). As a result, the variant was categorized as “likely pathogenic” according to the criteria and guidelines of the American College of Medical Genetics and Genomics (ACMG) (PM1, PM2, PP3, PP4).

Here, we also summarized all the *SORD* mutations reported in previous literatures (Table 2). A total of 101cases with 18 mutations were reported [4, 8, 19–29]. Among these, 73 patients carried the homozygous deletion variant c.757delG (p.A253Qfs*27), 27 cases in a compound heterozygous state of combination of c.757delG with another variant, one patient harboring a compound heterozygous variants of c.404 A > G and c.908 + 1G > C. Most of the variants in *SORD* are frameshift or splicing variants. Surprisingly, a case of a 24-year-old man diagnosed with juvenile amyotrophic lateral sclerosis (JALS) was documented. The patient was found to carry the homozygous c.757delG variant in the *SORD* gene [29]. The novel homozygous variant c.361G > C (p.Ala121Pro) detected in our patient was the second homozygous variant identified in the *SORD* gene related dHMN.

**Table 2:** Variants of the SORD gene previously reported in the literature

Variant 1	Variant 2	No. of cases	References
c.28 C > T; p.Leu10Phe	c.757delG; p.Ala253GlnfsTer27	1	Cortese et al., 2020
c.218 C > T; p.Ser73Leu	c.757delG; p.Ala253GlnfsTer27	1	Laššuthová et al., 2021
c.298 C > T; p.Arg100Te	c.757delG; p.Ala253GlnfsTer27	1	Cortese et al., 2020
c.316_425 + 165del; p.Cys106Ter	c.757delG; p.Ala253GlnfsTer27	1	Cortese et al., 2020
c.329G > C; p.Arg110Pro	c.757delG; p.Ala253GlnfsTer27	1	Cortese et al., 2020
c.404 A > G; p.His134Arg	c.908 + 1 G > C	1	Cortese et al., 2020
c.458 C > A; p.Ala153Asp	c.757delG; p.Ala253GlnfsTer27	12	Cortese et al., 2020; Frasquet et al., 2021; Laššuthová et al., 2021
c.503G > A; p.Gly168Asp	c.757delG; p.Ala253GlnfsTer27	1	Laššuthová et al., 2021
c.553G > A; p.Gly185Arg	c.757delG; p.Ala253GlnfsTer27	1	Laššuthová et al., 2021
c.625 C > T; p.Arg209Ter	c.757delG; p.Ala253GlnfsTer27	2	Grosz et al., 2022; Yuan et al., 2021
c.731 C > T; p.P244L	c.757delG; p.Ala253GlnfsTer27	1	Liu et al., 2021
c.757delG; p.Ala253GlnfsTer27	c.757delG; p.Ala253GlnfsTer27	73	Cortese et al., 2020; Frasquet et al., 2021; Dong et al., 2021; Wu et al., 2022; Yuan et al., 2021; Record et al., 2022; Laššuthová et al., 2021; Liu et al., 2021; Xie et al., 2020; Alluqmani et al., 2022; Bernard et al., 2022
c.776 C > T; p.A259V	c.757delG; p.Ala253GlnfsTer27	1	Liu et al., 2021
c.786 + 1 G > A	c.757delG; p.Ala253GlnfsTer27	1	Chen et al., 2022
c.851T > C; p.L284P	c.757delG; p.Ala253GlnfsTer27	1	Liu et al., 2021
c.895 C > T; p.Arg299Ter	c.757delG; p.Ala253GlnfsTer27	1	Cortese et al., 2020
c.964G > A; p.Val322Ilep	c.757delG; p.Ala253GlnfsTer27	1	Cortese et al., 2020

## Discussion

Even after conducting screenings for all known genetic mutations, a significant number of patients with hereditary neuropathies remain without a confirmed genetic diagnosis. In 2020, Cortese et al. reported that *SORD* was identified as a novel causative gene of recessive forms of dHMN and CMT2, with an estimated frequency of up to ∼ 10% in undiagnosed dHMN and CMT2 cases [8]. Our systemic literature review found 18 mutations from 101 affected individuals among different populations were identified to carry biallelic variants in *SORD*. Almost all described patients had the homozygous deletion variant c.757delG (p.A253Qfs*27) or a compound heterozygous state of a combination of c.757delG with another variant, besides one patient harboring a compound heterozygous for c.404 A > G and c.908 + 1G > C [19]. In the present study, the novel homozygous mutation c.361G > C (p.Ala121Pro) of *SORD* identified in our patient with dHMN was the second homozygous variant in *SORD* gene related dHMN.

The clinical manifestation of the patient was childhood-onset and symmetrical distal peripheral neuropathy. Physical examination showed brisk knee jerk and mild fasciculations in the thighs. Electrophysiological studies, including MNC, SNC, and EMG, revealed reduced compound muscle action potential (CMAP) amplitude in both lower limbs, normal sensory conductions, and chronic denervation in the upper and lower limbs. The characteristics of SORD-related CMT have been typically described as a length-dependent neuropathy, characterized by distal hereditary motor neuropathy (dHMN) or a predominantly motor presentation resembling CMT2. Unusual features were also observed, including mild hearing loss, dermographism, brisk reflexes, denervation, conduction block, and small fiber impairment on EMG [8, 26]. This may be attributed to diabetic mononeuropathy caused by high sorbitol levels involved in the pathogenesis.

*SORD* encodes a 357 amino acid protein and is widely expressed in mammalian tissues. SORD protein is sorbitol dehydrogenase involved in a two-step of polyol pathway. The initial step is the conversion of glucose to sorbitol by aldose reductase, followed by sorbitol dehydrogenase catalyzes sorbitol into fructose, which has been implicated in the pathophysiology of preclinical models of diabetic neuropathy [30, 31]. In the present study, a homozygous variant c.361G > C (p.Ala121Pro) of *SORD* gene were identified in a dHMN patient using whole exome sequencing. *In silico* pathogenicity prediction tools revealed that the mutation was categorized as “likely pathogenic” according to the criteria and guidelines of ACMG. Previous study found that patients with SORD mutations exhibited elevated fasting serum sorbitol levels, which resulted from decreased SORD enzyme levels and impaired enzyme function. The absence of *SORD* orthologues in Drosophila caused synaptic degeneration and motor impairment. It is suggested that loss-of-function of the enzyme and subsequent sorbitol accumulation was the mechanism of *SORD*-associated neuropathy [8]. A previous study demonstrated that aldose reductase inhibitors effectively reduced sorbitol accumulation in mouse models of diabetes and in humans [31]. This represents a promising therapeutic intervention for *SORD*-associated patients.

In conclusion, we reported one novel homozygous variant c.361G > C (p.Ala121Pro) of *SORD* identified in a Chinese patient with dHMN phenotype. This study expands the clinical and mutational spectrum of SORD-associated hereditary neuropathy. Further, these findings provide support for SORD being a disease-causing gene in dHMN, underscoring the significance of screening for SORD variants in patients with undiagnosed hereditary neuropathy patients. Enzyme therapy of the sorbitol metabolic pathway may be a promising therapeutic intervention for *SORD*-related neuropathy in the future.

## Data Availability

The datasets analyzed during the current study are available in the ClinVar database (https://submit.ncbi.nlm.nih.gov/subs/variation_clinvar/SUB14261784/. Submission ID: SUB14261784).
